# Pericardial metastases causing electrocardiographic changes: a case report

**DOI:** 10.1093/ehjcr/ytaf499

**Published:** 2025-10-04

**Authors:** Xianru Pan, Huijuan Zhang, Xinghui Li, Xiaomei Ma, Xing Wang

**Affiliations:** The First Clinical Medical College, Lanzhou University, No. 222 Tianshui South Road, Chengguan District, Lanzhou, Gansu 730000, China; The First Clinical Medical College, Gansu University of Chinese Medicine, No. 35 Dingxi East Road, Chengguan District, Lanzhou, Gansu 730000, China; The First Clinical Medical College, Lanzhou University, No. 222 Tianshui South Road, Chengguan District, Lanzhou, Gansu 730000, China; Department of Cardiology, Gansu Provincial People’s Hospital, No. 204 Donggang West Road, Chengguan District, Lanzhou, Gansu 730000, China; The First Clinical Medical College, Lanzhou University, No. 222 Tianshui South Road, Chengguan District, Lanzhou, Gansu 730000, China; The First Clinical Medical College, Lanzhou University, No. 222 Tianshui South Road, Chengguan District, Lanzhou, Gansu 730000, China

**Keywords:** Metastatic cardiac tumours, Pericardial metastasis, Electrocardiographic changes, Small-cell lung cancer, Case report

## Abstract

**Background:**

Owing to the non-specific clinical manifestations of cardiac metastases, these conditions frequently remain undetected until advanced stages, thus delaying optimal therapeutic intervention. Consequently, enhancing vigilance and the capability to identify early indicators are essential for facilitating timely diagnosis and effective management of cardiac metastases.

**Case summary:**

A 55-year-old Asian male presented with a 1-month history of intermittent abdominal distension, which had exacerbated in the preceding 2 days, accompanied by chest tightness and dyspnoea. An electrocardiogram (ECG) revealed sinus tachycardia (heart rate = 109 b.p.m.), a positive P terminal force in lead V1 (PTFV1) along with inadequate R wave progression in leads V1–V3, and partial T wave abnormalities. Cardiac colour Doppler ultrasound identified an intrapericardial mass. Subsequent investigations confirmed the diagnosis of small-cell lung carcinoma with pericardial metastasis. The patient received systemic chemotherapy with the EC regimen, completed two cycles. Follow-up ECG demonstrated sinus rhythm at a heart rate of 60 b.p.m. with some T wave changes in leads I, aVL, and V3–V6. Cardiac ultrasound indicated a significant reduction in intrapericardial lesions compared to prior findings, with concurrent improvement in symptoms of chest tightness and enhanced exercise tolerance.

**Discussion:**

Electrocardiography, particularly when showing dynamic changes, may provide clues for cardiac metastases, and its changes may demonstrate a significant correlation with pericardial metastatic tumour burden. This method potentially indicates treatment efficacy and facilitates the early detection of cardiac metastases.

Learning points

- Early pericardial metastatic tumours may induce electrocardiogram (ECG) alterations, including positive PTFV1, decreased T wave amplitudes in inferior leads, and abnormal R wave progression in precordial leads. These changes tend to normalize gradually with tumour reduction post-treatment.
- Cardiac metastases can disrupt myocardial cellular structure and function through multiple mechanisms, including mechanical compression, direct invasion, and inflammatory factor release. The ECG abnormalities represent a complex, multi-mechanism pathological process.
- For patients with malignant tumours, unexplained ECG variations should prompt careful consideration of potential cardiac metastasis. Electrocardiogram, as a convenient and accessible examination method, may provide early indications of cardiac metastatic involvement.

## Introduction

Pericardial metastases, defined as malignant neoplasms that disseminate to the pericardium via haematogenous or lymphatic routes, predominantly occur in individuals with advanced-stage malignancies, with lung cancer, breast cancer, lymphoma, melanoma, and gastrointestinal tumours being among the more common primary sources.^1^ The clinical presentation of these metastases lacks specificity and is closely associated with both tumour dimensions and the extent of tissue infiltration. Herein, we present a case of small-cell lung carcinoma with pericardial metastasis that demonstrated characteristic electrocardiographic alterations throughout the disease course and therapeutic intervention, suggesting the potential utility of electrocardiography as an initial screening method for cardiac metastasis.

## Summary figure

**Table:** 

Comparative analysis of patients’ electrocardiograms (ECGs), echocardiograms, and symptoms related to tumour cardiac metastases			
	Electrocardiographic changes	Echocardiographic changes	Related clinical symptoms
Initial hospitalization	T wave flattening in leads II, III, and aVF (≤0.1 mV), positive PTFV1, poor R wave progression in leads V1–V3 (≤0.1 mV), sinus tachycardia	A 62 × 26 mm space-occupying lesion within the pericardium. Left ventricular wall motion abnormality	Chest tightness and shortness of breath, lower extremity oedema
After one cycle of treatment	T waves in leads II, III, and aVF became upright; the heart rate remained elevated with frequent premature ventricular complexes, and changes in the chest leads were insignificant, sinus tachycardia	A 47 × 28 mm space-occupying lesion within the pericardium. Right atrial compression	Reduction in lower extremity oedema severity
After two cycles of treatment	T waves in leads II, III, and aVF were notably upright (0.1–0.2 mV), the previously positive P wave terminal force in V1 (PTFV1) had normalized, and there was a significant increase in R wave amplitude in leads V1–V3 compared to earlier recordings (0.5–1.6 mV)	A 12 × 5 mm space-occupying lesion within the pericardium. Relief of right atrial compression	Activity endurance is significantly higher than before

## Case presentation

A 55-year-old Asian male presented with a 1-month history of intermittent abdominal distension that had exacerbated over the preceding 2 days, accompanied by chest tightness and dyspnoea. He had no significant past medical history. Cardiac ultrasound revealed enlargement of the left atrium (LA) and left ventricle (LV), along with reduced left ventricular systolic function (left ventricular ejection fraction: 15%). He was subsequently admitted to the cardiology department with a diagnosis of dilated cardiomyopathy and heart failure. Upon admission, physical examination revealed a body temperature of 36.4°C, pulse rate of 101 b.p.m., respiratory rate of 20 breaths/minute, and blood pressure of 107/76 mmHg. The patient was alert, with coarse breath sounds bilaterally and crackles at the lung bases. Cardiac examination demonstrated an enlarged cardiac silhouette, a regular rhythm with a rate of 101 b.p.m., diminished heart sounds, and an audible third heart sound (S3) at the apex. No pathological murmurs were detected. The abdomen was flat with mild tenderness in the upper quadrants. The liver was palpable 3 cm below the right costal margin, characterized by a rounded edge and smooth surface, without significant nodules on palpation. The spleen was not palpable, and shifting dullness was positive. No abdominal vascular murmurs were detected, and bilateral lower extremity pitting oedema was present. Laboratory examinations revealed elevated levels of creatine kinase-MB (39.40 U/L; reference range: <25 U/L), lactate dehydrogenase (318.36 U/L; reference range: 120–250 U/L), homocysteine (28.96 μmol/L; reference range: <15 μmol/L), and N-terminal pro-B-type natriuretic peptide (3607 pg/mL; reference range: <125 pg/mL). High-sensitivity troponin I was 0.02 ng/mL (reference range: <0.026 ng/mL), C-reactive protein was 5.72 mg/L (reference range: 0–6 mg/L), and white blood cell count was 6.2 × 10^9^/L (reference range: 3.5–9.5 × 10^9^/L).

**Figure 1:**
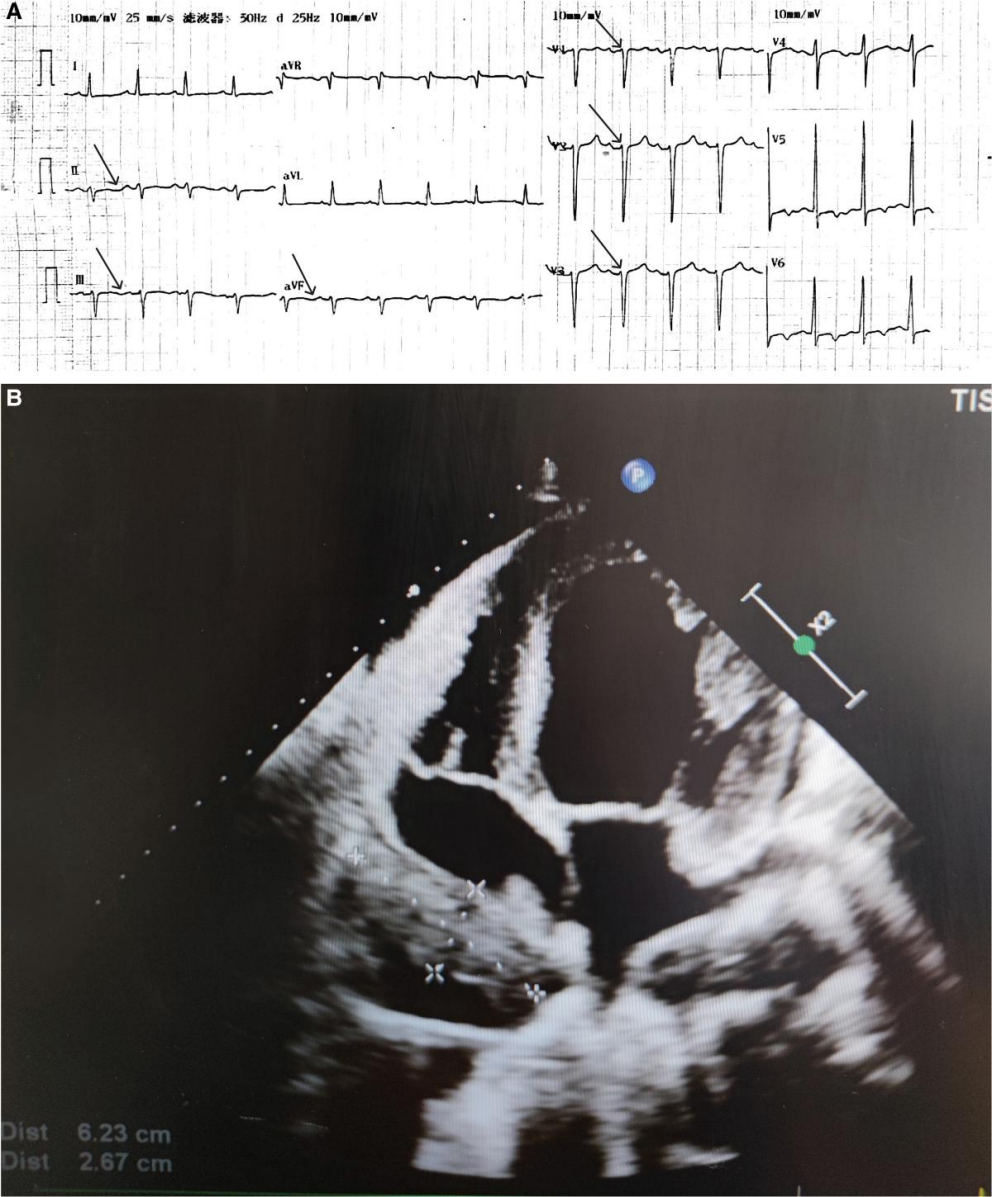
(*A*) Twelve-lead electrocardiogram recorded upon the patient’s admission to the hospital. (*B*) Echocardiographic images obtained during the patient’s initial hospital admission.

The ECG demonstrated sinus tachycardia (109 b.p.m.), positive P-terminal force in lead V1 (PTFV1), poor R wave progression in leads V1-V3, and non-specific T wave abnormalities (*Figure 1A*). Echocardiography revealed a 62 × 26 mm intrapericardial mass causing right atrial compression, abnormal left ventricular wall motion, enlargement of the left atrial cavity (LA: 40 × 55 mm), left ventricular enlargement (LV: 54 × 77 mm), dilation of the inferior vena cava, and slight left ventricular hypertrophy. Pulmonary hypertension was evident (systolic pulmonary artery pressure: 45 mmHg), accompanied by minimal pericardial effusion. Left ventricular diastolic function was graded as Type II dysfunction, and left ventricular systolic function was reduced with an ejection fraction of 34% calculated using Teichholz’s method (*Figure 1B*). To further characterize these findings, contrast echocardiography and cardiac magnetic resonance imaging (CMRI) of the left heart were performed. Based on the imaging results, the mass was determined to originate from neoplastic lesions of the pericardium. Subsequently, a cardiothoracic surgery consultation was obtained to evaluate the suspected malignant pericardial tumour. To determine the extent of the disease, additional investigations—including chest computed tomography (CT), tumour marker analysis, and positron emission tomography/CT—were conducted to assess for potential metastatic disease. To establish the primary malignancy, a mediastinal lymph node biopsy was performed. Integration of clinical findings with immunohistochemical staining results confirmed the diagnosis of extensive-stage small-cell lung cancer (SCLC). Based on the patient’s clinical status and in accordance with treatment guidelines, an etoposide-carboplatin (EC) regimen was initiated. The chemotherapy protocol consisted of etoposide 100 mg daily on Days 1–5 and carboplatin 400 mg on Day 1 of each cycle. The patient was discharged following the resolution of chest tightness and dyspnoea.

**Figure 2:**
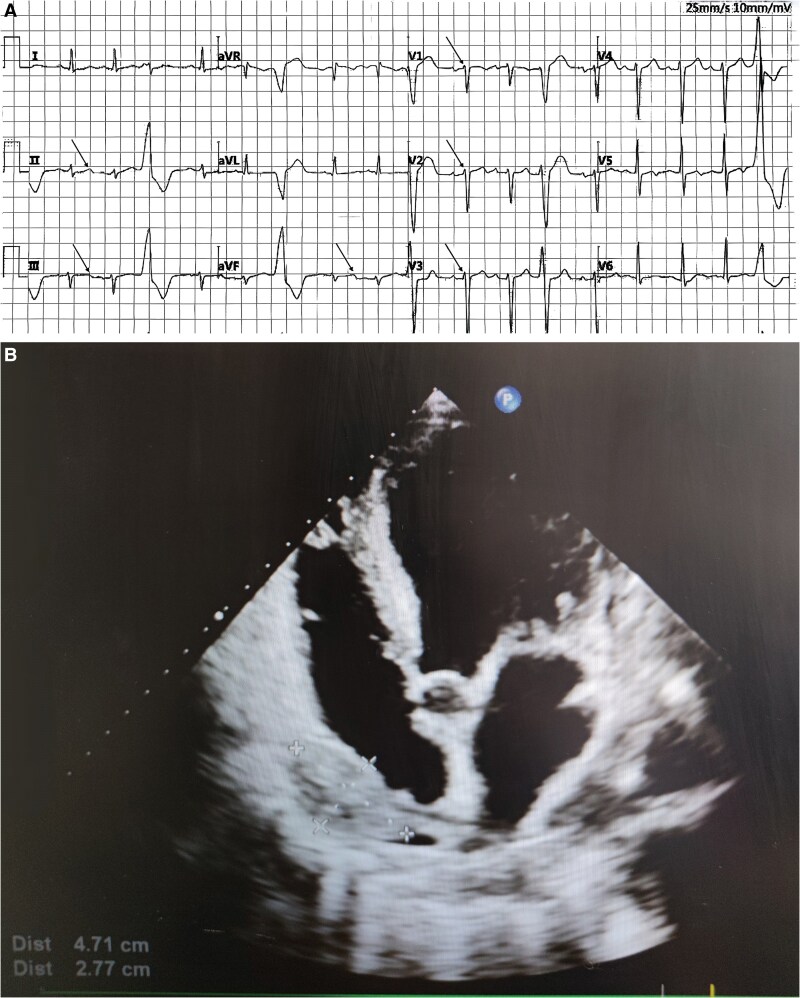
(*A*) Twelve-lead electrocardiogram following the patient’s first treatment session. (*B*) Echocardiographic images obtained after the patient’s first treatment session.

**Figure 3:**
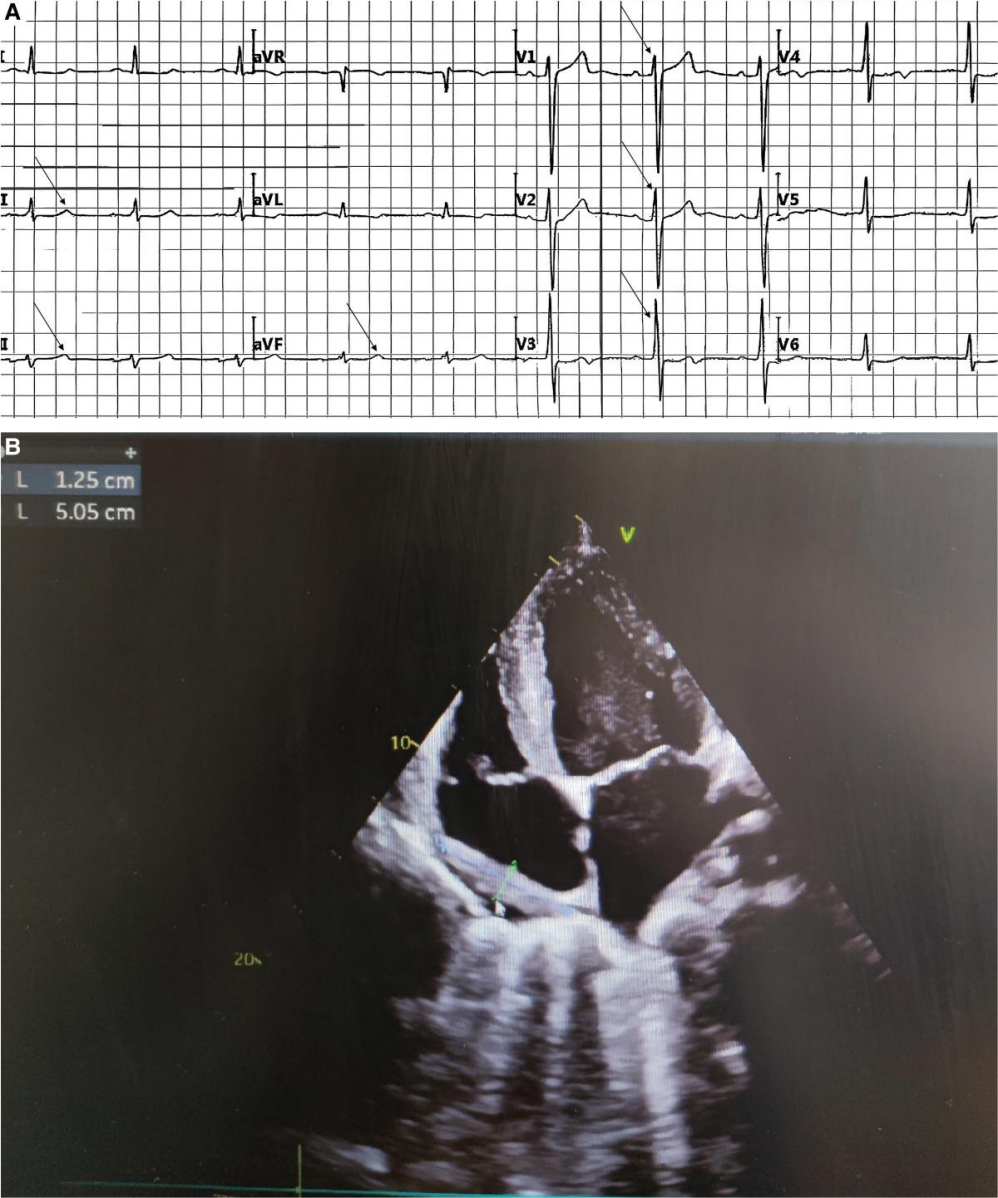
(*A*) Twelve-lead electrocardiogram following the patient’s second treatment session. (*B*) Echocardiographic images obtained after the patient’s second treatment session.

At the 1-month follow-up assessment, laboratory results showed creatine kinase-MB at 19.49 U/L, lactate dehydrogenase at 215.61 U/L, NT-proBNP at 2923 pg/mL, high-sensitivity troponin I at 0.0080 ng/mL, C-reactive protein at 15.57 mg/L, and a white blood cell count of 7.5 × 10^9^/L. Electrocardiography revealed sinus tachycardia with a heart rate of 108 b.p.m., frequent ventricular premature triple rhythm, positive PTFV1, poor R wave progression in the anterior precordial leads, and T wave abnormalities (*Figure 2A*). Cardiac ultrasound demonstrated a pericardial effusion measuring 47 × 28 mm with displacement and right atrial compression, along with abnormal left ventricular wall motion, enlargement of the LA (46 × 55 mm) and LV (53 × 86 mm), slight left ventricular hypertrophy, minimal pericardial effusion, Grade II left ventricular diastolic dysfunction, and reduced systolic function with an ejection fraction of 28% using Teichholz’s method (*Figure 2B*). The pericardial mass appeared smaller compared to the prior examination. Following optimization of cardiac function and management of arrhythmias, the patient underwent the second cycle of chemotherapy with the same regimen. By the 3-month follow-up, the third cycle of chemotherapy had been completed. Laboratory tests now showed improved values including creatine kinase-MB at 15.42 U/L, lactate dehydrogenase at 191.81 U/L, NT-proBNP at 1250 pg/mL, high-sensitivity troponin at 0.0040 ng/mL, C-reactive protein at 13.11 mg/L, and leukopenia with a white blood cell count of 1.5 × 10^9^/L. The ECG displayed sinus rhythm at 60 b.p.m. with T wave changes observed in leads I, aVL, and V3–V6 (*Figure 3A*). Cardiac ultrasound revealed resolution of right atrial compression with a smaller pericardial effusion measuring 12 × 5 mm. An incidental ostium secundum atrial septal defect with left-to-right shunting was identified. The LA remained enlarged (50 × 58 mm) along with left ventricular dimensions (60 × 91 mm), and Grade I diastolic dysfunction was noted. Left ventricular systolic function had improved, with an ejection fraction of 44% by Teichholz’s method and only a trace of pericardial effusion was present (*Figure 3B*). With clinical improvement achieved after treatment, the patient was discharged and scheduled for outpatient follow-up.

## Discussion

Cardiac space-occupying lesions are conventionally classified into three principal categories: neoplasms, thrombi, and vegetations. Among neoplastic lesions, a fundamental distinction exists between primary and metastatic entities. Primary cardiac neoplasms represent a rare entity, with an estimated incidence ranging from 2 to 30 cases per 100 000 individuals.^2^ Approximately 80% of primary cardiac neoplasms are histologically benign, with myxomas constituting the predominant subtype, while the remaining 20% exhibit malignant characteristics. In contrast, metastatic cardiac involvement occurs with substantially higher frequency, approximately 100-fold that of primary cardiac neoplasms. Virtually, any malignant neoplasm harbours the potential for cardiac metastasis. The mechanisms of metastatic dissemination include direct extension, haematogenous spread, lymphatic infiltration, and intracaval extension via the inferior vena cava. The most common primary malignancies associated with cardiac metastases include bronchogenic carcinoma, mammary carcinoma, oesophageal carcinoma, haematologic malignancies, and melanoma.^3,4^ Primary and metastatic cardiac tumours exhibit somewhat distinct but generally non-specific clinical manifestations. The symptomatology of primary cardiac neoplasms typically correlates with their intrinsic pathophysiological characteristics, manifesting as heart failure, arrhythmias, and valvular dysfunction. Distinctively, myxomas may generate a characteristic ‘tumour plop’ sound during cardiac auscultation. The clinical presentation of metastatic cardiac involvement varies according to tumour histology, anatomical location, metastatic potential, and pattern of cardiac infiltration. The pericardium represents the most frequently involved cardiac structure, followed by the epicardium, myocardium, and endocardium.^5^ Pericardial involvement predominantly manifests as pericardial effusion, which may progress to cardiac tamponade with consequent haemodynamic compromise. Electrocardiographic features frequently include low-voltage QRS complexes, electrical alternans, and ST-T wave abnormalities. Epicardial involvement typically results in localized cardiomyopathy and extracardiac compressive phenomena, with electrocardiographic manifestations commonly including regional ST-segment alterations. Myocardial involvement directly compromises myocardial cellular architecture and function, resulting in conduction system disturbances. Electrocardiographic findings in this context encompass various arrhythmias, conduction blocks, and ischaemia-like alterations. Endocardial involvement, though least prevalent, may produce intracavitary space-occupying effects, resulting in intracardiac flow obstruction, valvular dysfunction, and enhanced thromboembolism risk. Electrocardiography frequently demonstrates localized pathological Q waves or ST-T wave abnormalities. Myocardial and endocardial invasion typically portend a less favourable prognosis compared with isolated pericardial involvement, attributable to direct impairment of myocardial contractility and disruption of electrical conduction pathways. In the present case, tumour burden reduction was accompanied by progressive resolution of electrocardiographic abnormalities, a suggesting favourable therapeutic response. The reversibility of electrocardiographic alterations may serve as a surrogate marker for treatment efficacy assessment.

Histopathological sampling of cardiac masses presents considerable technical challenges; consequently, diagnosis frequently relies on characteristic imaging features. Echocardiography, cardiac CT (CCT), and CMRI each contribute uniquely to the diagnostic evaluation of cardiac space-occupying lesions. Specifically, echocardiography enables real-time assessment of lesion dimensions, anatomical location, and haemodynamic consequences. Conversely, CCT and CMRI excel in delineating spatial relationships and providing tissue characterization of cardiac lesions.^6^ In contrast to the aforementioned advanced imaging modalities, electrocardiography (ECG) represents a readily accessible, non-invasive, and cost-effective diagnostic tool requiring minimal technical infrastructure. Its ubiquitous availability across diverse healthcare settings—from tertiary referral centres to resource-limited facilities—ensures widespread accessibility, establishing ECG as an economically viable initial screening approach. While electrocardiography cannot directly visualize neoplastic lesions, it provides valuable indirect evidence of tumour-related cardiac effects, including myocardial ischaemia, arrhythmogenesis, and ventricular hypertrophy. Notably, in patients exhibiting ischaemic ST-T segment abnormalities without established coronary artery disease, these electrocardiographic findings may constitute important diagnostic clues suggesting potential cardiac metastatic involvement.

Initial electrocardiographic evaluation revealed sinus tachycardia, flattened T waves in the inferior leads (II, III, and aVF), and poor R wave progression in the anterior precordial leads (V1–V3). Following 1 month of therapeutic intervention, T waves in the inferior leads normalized; however, persistent tachycardia was observed, accompanied by frequent premature ventricular contractions, with minimal alterations in the precordial lead morphology. At the 2-month follow-up assessment, heart rate demonstrated significant reduction compared with previous evaluations, T waves in the inferior leads exhibited pronounced upright configuration, the previously documented positive P-terminal force in lead V1 (PTFV1) abnormality resolved, and R wave amplitudes in leads V1–V3 showed substantial augmentation. Serial echocardiographic assessments demonstrated progressive diminution of the pericardial mass with concomitant resolution of cardiac compression, correlating with clinical improvement. The term PTFV1 refers to the product of the amplitude and duration of the negative deflection of the P wave in lead V1 on an ECG. Also known as the Morris index, an abnormal PTFV1 may indicate impaired atrial function or abnormalities in the atrial conduction system.^7^ In the present case, echocardiographic evaluation demonstrated left atrial enlargement and an atrial septal defect, both structural abnormalities potentially contributing to PTFV1 alterations. Nevertheless, these anatomical abnormalities persisted and progressively intensified across three consecutive echocardiographic evaluations. Conversely, PTFV1 normalized dynamically following reduction in tumour burden and subsequent alleviation of right atrial compression. This temporal correlation suggests that the PTFV1 abnormality predominantly reflected tumour-induced impairment of right atrial geometry and atrial conduction system function rather than chronic left atrial enlargement or the interatrial septal defect. The flattened T waves observed in the inferior leads may result from various pathophysiological processes, including myocardial ischaemia, myocarditis, pericarditis, electrolyte disturbances, or pharmacological effects.^8^ In the current case, the initial modest elevation of cardiac biomarkers (creatine kinase-MB and high-sensitivity troponin) followed by their gradual normalization did not correspond with the characteristic temporal evolution of acute myocardial infarction or sustained myocardial ischaemia. The initial elevation and subsequent reduction in N-terminal pro-B-type natriuretic peptide (NT-proBNP) and lactate dehydrogenase (LDH) levels paralleled changes in cardiac haemodynamic burden and tumour mass. Despite radiographic evidence of pericardial metastasis, the absence of diffuse ST-segment elevation characteristic of pericarditis and the non-persistent pattern of C-reactive protein dynamics argue against significant inflammatory pericardial involvement. Given the temporal concordance between T wave normalization on electrocardiography and the echocardiographically documented reduction in tumour dimensions with subsequent alleviation of cardiac compression, we propose that the most plausible aetiology of the flattened T waves in the inferior leads was direct mechanical compression or local effects of the pericardial metastatic lesion on the subepicardial myocardium, disrupting normal ventricular repolarization processes, rather than atypical coronary ischaemia or primary pericardial inflammation.

Furthermore, tumour-induced right heart compression produced corresponding electrocardiographic alterations predominantly affecting the inferior and right precordial leads. Consequently, serial electrocardiographic changes can function as indirect indicators for monitoring tumour burden dynamics and approximating anatomical localization. The detection of an atrial septal defect at the third evaluation likely occurred because earlier right atrial compression obscured this pre-existing anatomical anomaly; only following the resolution of compression and restoration of right atrial architecture did the defect become echocardiographically apparent. Li and Sun^9^ reported a case of a patient with SCLC undergoing standard chemotherapy who presented 2 years after initial diagnosis with chest discomfort and dyspnoea. Electrocardiography demonstrated widespread concave ST-segment elevation in precordial leads, initially suggestive of acute myocardial infarction; however, serial cardiac biomarkers remained within normal limits, without evidence of persistent ST-segment elevation or pathological Q wave formation. Subsequent thoracic CT and echocardiography confirmed cardiac metastasis with pericardial effusion, definitively excluding acute coronary syndrome. This report highlighted that subtle early electrocardiographic alterations may provide indirect evidence facilitating earlier detection of cardiac metastases, with electrocardiography potentially serving as the sole initial indicator despite the absence of demonstrable imaging abnormalities.

Cardiac metastases influence myocardial electrophysiological properties through multiple pathophysiological mechanisms. Pericardial tumour infiltration frequently precipitates pericardial effusion, while solid tumour mass exerts mechanical compression on adjacent myocardial tissue, potentially disrupting normal ventricular repolarization processes; these factors constitute the primary mechanisms underlying T wave abnormalities. The attenuation of R wave voltage predominantly results from the electrical insulating effect of pericardial effusion and impaired myocardial contractile function secondary to tumour-induced compression, which collectively compromise normal electrical impulse propagation between cardiomyocytes, manifesting as reduced QRS complex amplitude.^10,11^ Additionally, cardiac metastases may directly infiltrate the myocardium, causing architectural disruption of myocardial tissue and inducing localized inflammatory responses through the release of pro-inflammatory cytokines, including tumour necrosis factor-alpha and interleukin-6, which subsequently trigger inflammatory cascades and promote cardiomyocyte apoptosis. Furthermore, the mass effect of tumour infiltration may compress coronary arteries and cardiac chambers, inducing regional haemodynamic disturbances and ischaemic myocardial injury. Chemotherapeutic agents administered for malignancy management may further contribute to cardiotoxicity through multiple mechanisms, including induction of cardiomyocyte apoptosis, generation of reactive oxygen species, and disruption of mitochondrial function.^12^ Patients with advanced malignancies frequently exhibit varying degrees of electrolyte imbalance, particularly involving potassium and calcium homeostasis, which alter cardiomyocyte membrane potential and adversely affect cellular excitability and electrical conduction. In this case, the improvement in cardiac electrophysiological parameters following sequential therapeutic interventions closely correlated with reduced tumour burden and decreased levels of inflammatory mediators, indicating the predominant role of tumour-related factors in myocardial electrophysiological abnormalities. Simultaneously, potential chemotherapy-induced cardiotoxicity and other systemic factors collectively contribute to the complex myocardial pathophysiology, resulting in the observed electrocardiographic abnormalities.

This case emphasizes the importance of heightened clinical vigilance when encountering unexplained electrocardiographic abnormalities—particularly T wave alterations and diminished R wave amplitude—in patients presenting with cancer cachexia or other systemic manifestations of malignancy. Persistent asymptomatic electrocardiographic alterations may represent early indicators of cardiac metastatic involvement. In patients with established malignancy, the emergence of dynamic electrocardiographic changes should prompt comprehensive evaluation for potential cardiac metastases. Electrocardiography, as a non-invasive, cost-effective, and readily accessible diagnostic modality, can be utilized as a primary screening tool for surveillance in patients at elevated risk for cardiac metastasis, potentially facilitating early detection and timely therapeutic intervention.

## Data Availability

The data underlying this article are available to use for all readers.

## Lead author biography

I am Xianru Pan, currently being educated at Lanzhou University, one of the prestigious institutions in China. I am pursuing a master’s degree with a focus on cardiology. My research interests centre around cardiovascular diseases, aiming to contribute to advancing knowledge and therapeutic strategies in this critical area of medicine.

## Notes

**Consent:** Informed consent was obtained from the patient in accordance with the Committee on Publication Ethics (COPE) guidelines.

**Funding:** None declared.
